# Efficient retina formation requires suppression of both Activin and BMP signaling pathways in pluripotent cells

**DOI:** 10.1242/bio.20149977

**Published:** 2015-03-06

**Authors:** Kimberly A. Wong, Michael Trembley, Syafiq Abd Wahab, Andrea S. Viczian

**Affiliations:** 1 Department of Ophthalmology, SUNY Upstate Medical University, Syracuse, NY 13210, USA; 2 Department of Neuroscience and Physiology, SUNY Upstate Medical University, Syracuse, NY 13210, USA; 3 The Center for Vision Research, SUNY Eye Institute, Upstate Medical University, Syracuse, NY 13210, USA; 4 Department of Pharmacology and Physiology, Aab Cardiovascular Research Institute, University of Rochester School of Medicine and Dentistry, Rochester, NY 14642, USA; 5 Department of Molecular Biology, Weill Cornell Graduate School of Medical Sciences, New York, NY 10021, USA

**Keywords:** Noggin, Retina, *Xenopus*, Animal cap, BMP, Activin, R-SMAD, SMAD1, SMAD2, SB431542, Dorsomorphin

## Abstract

Retina formation requires the correct spatiotemporal patterning of key regulatory factors. While it is known that repression of several signaling pathways lead to specification of retinal fates, addition of only Noggin, a known BMP antagonist, can convert pluripotent *Xenopus laevis* animal cap cells to functional retinal cells. The aim of this study is to determine the intracellular molecular events that occur during this conversion. Surprisingly, blocking BMP signaling alone failed to mimic Noggin treatment. Overexpressing Noggin in pluripotent cells resulted in a concentration-dependent suppression of both Smad1 and Smad2 phosphorylation, which act downstream of BMP and Activin signaling, respectively. This caused a decrease in downstream targets: endothelial marker, *xk81*, and mesodermal marker, *xbra*. We treated pluripotent cells with dominant-negative receptors or the chemical inhibitors, dorsomorphin and SB431542, which each target either the BMP or Activin signaling pathway. We determined the effect of these treatments on retina formation using the Animal Cap Transplant (ACT) assay; in which treated pluripotent cells were transplanted into the eye field of host embryos. We found that inhibition of Activin signaling, in the presence of BMP signaling inhibition, promotes efficient retinal specification in *Xenopus* tissue, mimicking the affect of adding Noggin alone. In whole embryos, we found that the eye field marker, *rax*, expanded when adding both dominant-negative Smad1 and Smad2, as did treating the cells with both dorsomorphin and SB431542. Future studies could translate these findings to a mammalian culture assay, in order to more efficiently produce retinal cells in culture.

## Introduction

Early experiments in amphibians first showed that neural tissue was specified by the inhibition of BMP signals by proteins secreted by the Spemann organizing center in the dorsal mesoderm (reviewed in Weinstein and Hemmati-Brivanlou, 1999; Harland, 2000; De Robertis and Kuroda, 2004). Since that time, a second signaling center has been identified in the dorsal blastula, called the blastula Chordin- and Noggin-expressing (BCNE) region (Kuroda et al., 2004). Together these regions ensure neural induction by modulating the activity of the bone morphogenetic protein (BMP) and Wnt signaling pathways to form dorsal-to-ventral and anterior-posterior concentration gradients that pattern the developing embryo (reviewed in De Robertis and Kuroda, 2004). The secreted protein, Noggin, was one of the first organizer-specific neural inducers to be identified and cloned (Lamb et al., 1993; Smith et al., 1993; Zimmerman et al., 1996). Other secreted molecules, including Chordin, Follistatin, and Cerberus, were later identified and collectively these molecules became known as the BMP antagonists. These findings support the idea of a “default model” of neural induction in which BMP signaling must be repressed to allow neural cells to form.

While the extrinsic signaling mechanisms regulating neural induction have been well studied, very little is known about the intrinsic mechanisms responsible for retinal progenitor cell specification. Retinal development begins very early in the blastula and later in the anterior-most region of the neural plate called the eye field (Zaghloul et al., 2005). Work in *Xenopus* has suggested that Noggin acts as a morphogen to upregulate a network of eye field transcription factors (EFTFs) that are both required and sufficient to drive a retinal progenitor cell fate in pluripotent ectodermal explants, also called animal caps (Zuber et al., 2003; Lan et al., 2009; Viczian et al., 2009). Pluripotent cells exposed to higher concentrations of Noggin from gastrula to neurula stage (stages 9 to 15, respectively) formed eye-like structures when transplanted to the flank and functional eyes when transplanted to the endogenous eye field of an age-matched host embryo (Viczian et al., 2009; Viczian and Zuber, 2014). These results suggest that Noggin is sufficient to specify functional retinal cells, but the intrinsic signaling mechanism driving this phenomenon remains unknown.

All the previous work in the field points to Noggin severely inhibiting BMP signaling at higher concentrations to illicit retinal formation. However, it was recently discovered that Noggin may also bind Activin ligands (Bayramov et al., 2011). Activin signaling was largely studied for its role in mesoderm specification, but recently, work by Chang and Harland moves away from this “default model” of neural specification to suggest that the dual inhibition of BMP-Smad1 and Activin/TGFβ-Smad2 signaling is required for efficient neural induction (Chang and Harland, 2007). Yet, it remained to be tested *in vivo* whether Activin inhibition affects retina formation. Activins are another member of the TGFβ family of signaling molecules, which share a signaling pathway with TGFβ and Nodal. To signal, BMP, Activin, or TGFβ ligands bind to the extracellular domain of two single membrane spanning receptors, referred to as type I and type II. Upon ligand binding, the two receptors complex and the type II receptor phosphorylates the type I receptor tail to activate Smad-mediated gene transcription (reviewed in Lin et al., 2006). In this study, we will refer to the Activin/TGFβ/Nodal pathway as the Activin pathway, since Noggin was shown to bind this ligand.

To address these questions and better understand the role of the BMP and Activin inhibition in retinal specification, we inhibited these pathways by expressing dominant-negative receptors and dominant-negative Smads, and treating with known pharmacological agents. Using the Animal Cap Transplant assay as previously described (Viczian et al., 2009; Viczian and Zuber, 2010), we assayed the ability for these treatments to drive retina formation from pluripotent cells. Our data suggest that Noggin can inhibit the activity of the Smad1/5/8 and Smad2/3 (also called receptor regulated Smads, or R-Smads) downstream of both the BMP and Activin pathways, respectively. Furthermore, dual inhibition of both pathways is required to drive the efficient formation of retina from *ex vivo* pluripotent cells, and results in *in vivo* eye field expansion. For the first time, we have found that reduction of the Activin pathway, in conjunction with BMP repression, is necessary for efficient retina formation.

## Materials and Methods

### Animals and transplantation

*Xenopus laevis* embryos were obtained through *in vitro* fertilization. This procedure, and others described here, were done following IACUC approved protocols. Embryos were staged according to Nieuwkoop and Faber (Nieuwkoop and Faber, 1994). For animal cap isolation, YFP RNA (500 pg) and experimental RNA were injected into both cells of a two-cell stage embryo. Concentration of RNA indicated was the amount injected in each cell. Animal caps were removed at stage 8.5–9 using a cauterizing tip (Protech International Inc., Boerne, Texas), and cultured (with or without chemical inhibitors in 0.7× MMR) to stage 15 for protein and RT-PCR analysis or used for transplantation, as described previously (Viczian and Zuber, 2010). Stock solutions of the chemical inhibitors, SB208350 (10 mM catalog number 559389; Millipore, Billerica, MA, USA) and dorsomorphin (5 mM; catalog number P5499; SIGMA, St. Louis, MO), were made by resuspending in sterile dimethyl sulfoxide (SIGMA); while 10 mM SB431542 stock was obtained (catalog number 04-0010-05; Stemgent, Cambridge, MA). The Animal Cap Transplant assay was performed on at least 17 embryos per biological replicate. Retinal tissue was scored positive when transplanted cells (YFP+) co-stained for XAP2 (rod photoreceptor marker).

### Immunohistochemistry and antibodies

Tadpoles were fixed, cryostat sectioned, immunostained using antibody concentrations described in supplementary material Table S1, and visualized at stage 41–43, as previously described (Viczian et al., 2003; Viczian et al., 2009).

### DNA constructs and PCR amplification

Xbmp4-b was PCR amplified from stage 20 *Xenopus* embryos and flanked by XhoI and XbaI restriction sites using the following primers: forward 5′-CTCGAGTTGTGTCCAACATTGGCTGT-3′ and reverse 5′-TCTAGAGGAAAGAAGTCCAGCCGTTA-3′. Xsmad2 was PCR amplified from stage 15 embryos and flanked by BstBI and Xhol restriction sites using the following primers: forward 5′-TTCGAAAACATGTCGTCCATCTTGCCTTTCACC-3′ and reverse 5′-CTCGAGATTAGGACATGCTTGAGCAGCGGAC-3′. PCR products were then cloned into pCS2+ and pCS2R vectors at the respective sites. Xsmad2-P445H was cloned by inducing a point mutation at amino acid 445 (CCT to CAT, Eppert et al., 1996) using the QuikChange II Site Directed Mutagenesis Kit (Agilent Technologies, Santa Clara, CA) using the following mutagenesis primers: forward 5′-GAGCTTCACCTGAATGGACACTTGCAGTGGTTGGACAAAG-3′ and reverse 5′-CTTTGTCCAACCACTGCAAGTGTCCATTCAGGTGAAGCTC-3′. A table of constructs made in this study or obtained from other sources can be found in supplementary material Table S2. All capped mRNA was synthesized from linearized DNA using the SP6 mMessage Machine kit (Life Technologies, Grand Island, NY). QRT-PCR standards were PCR amplified with appropriate primers and TA cloned into the pGEMTez vector. Sequence analysis on several clones verified primer pairs amplified the correct product.

### Quantitative and semi-quantitative reverse transcription PCR

Total RNA was isolated from ten stage 15 animal caps using RNAzol RT reagent (Molecular Research Center, Cincinnati, OH) according to manufacturer instructions. First strand synthesis of cDNA and subsequent RT-PCR was performed as described (Zuber et al., 2003) using MMLV Reverse Transcriptase (Promega, Madison, WI) and random hexamers, and EconoTaq DNA polymerase (Lucigen, Middleton, WI). A list of primers and respective RT-PCR cycling conditions can be found in supplementary material Table S3. PCR products were subcloned and sequenced to ensure fidelity to their target sequence. Relative gene expression was determined with respect to (wrt) YFP or Noggin using ImageLab (ChemiDoc XRS+, Bio-Rad, Hercules, CA), and normalized to *h4*. Quantitative real time PCR was conducted using a BioRad CRX384 Touch Real-Time PCR Detection System with LightCycler 480 SYBR Green I Master Mix (catalog number 04707516001; Roche, Indianapolis, IN). The PCR was run at 95°C for 3 min, then cycled 50 times through the following steps: 95°C, 15 seconds, 60°C, 15 seconds, 72°C, 15 seconds. Melting curves were obtained for each PCR run at 60° to 95°C. Absolute quantification was performed by standard curve method using PCR product-containing plasmids listed in supplementary material Table S2.

### Western blotting

Stage 15 animal caps were lysed in buffer containing 1% NP-40, 10 mM HEPES (pH 8.0), 150 mM NaCl, supplemented with cOmplete EDTA-free protease and PhosStop phosphatase inhibitor cocktail tablets (Roche). Using standard protocols, 20–75 µg of total protein was loaded per lane, separated by SDS-PAGE, and transferred to PVDF membrane. Membranes were blocked and stained as per antibody specifications (list of antibodies in supplementary material Table S1). Relative levels of target proteins were determined wrt YFP or Noggin using ImageJ (http://rsbweb.nih.gov/ij/), ImageLab, or Odyssey CLx system (LI-COR Biotechnology, Lincoln, Nebraska), and normalized to relative levels of β-actin.

### Whole mount *in situ* hybridization

Dioxigenin-labelled antisense RNA probes were generated from the 3′-untranslated region of xBMPrIIb (NCBI accession number NM_001088190.1) and xstk2 (NCBI accession number NM_001088010.1) using T7 or SP6 RNA Polymerase Plus enzymes (Life Technologies). β-Gal staining and whole mount *in situ* hybridization was performed for *rax* as described previously (Zuber et al., 2003; Viczian et al., 2006). Areas measured using ImageJ.

### Statistical tests

All statistical analysis was conducted on Prism software (6.0c) using an ordinary or repeated measures one-way ANOVA test, with a Tukey's multiple comparisons test unless otherwise indicated. Statistical significance was determined by p<0.05. All graphs depict the mean±s.e.m. normalized to YFP or Nog control (wrt, with respect to). Significance denoted by ns (not significant), *p<0.05, **p<0.01, or ***p<0.001. ‘*n*’, number of animals; ‘*N*’, number of biological replicates.

## Results

### Inhibition of canonical, but not non-canonical, BMP signaling can induce retinal formation

**Fig. 1.**
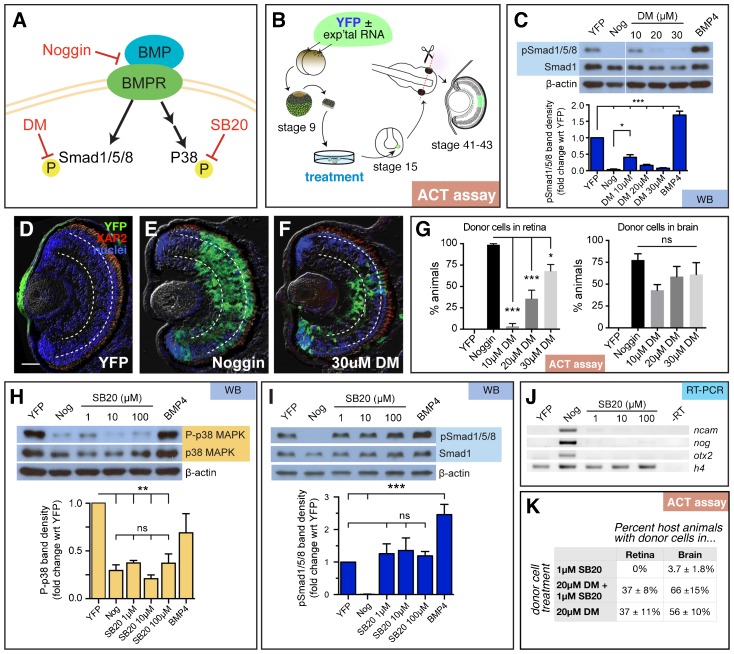
Repression of canonical and/or non-canonical BMP signaling fails to replicate the retina-inducing efficiency of Noggin. (A) Schematic of the canonical and non-canonical BMP pathways and the downstream signaling molecules, Smad1/5/8 and p38 MAPK, respectively. Small molecule inhibitors dorsomorphin (DM) and SB203580 (SB20) were used to specifically inhibit canonical and non-canonical signaling, respectively. (B) Diagram of experimental design for animal cap transplant (ACT) assay. YFP RNA with and without experimental (exp'tal) RNA was injected into both cells of a two-cell stage embryo. The animal cap was removed from the blastula (stage 9) and cultured until sibling embryos formed a neural plate (stage 15). Part of the animal cap was then transplanted into the eye field of a host embryo, which was grown until the eye differentiated (stages 41 to 43). Cryostat sections were analyzed for the presence of YFP+ transplanted cells. (C–G) Analysis of canonical signaling pathway. (C) Western blots were used to detect pSmad1/5/8, Smad1, and β-actin in stage 15 animal caps treated with DM. Treatment with 20 and 30 µM of DM is sufficient to suppress pSmad1/5/8 as efficiently as Noggin (Nog). (D–F) Representative images of transplanted cells in the retina. Treating animal caps with 30 µM of DM drives retinal specification in only 75% of embryos. Scale bars, 50 µm. Dashed lines lie on outer and inner plexiform layers, separating the three retinal layers. (G) The number of animals with transplanted cells in the eye were identified by scoring cryostat sections stained for YFP (green), rod photoreceptor marker, XAP2 (red), and DAPI (blue). Quantification of retinal integration efficiency, depicted as % of animals with YFP+ donor cells in the retina or brain. YFP, *n* = 44, Nog, *n* = 90; 10 µM DM, *n* = 46; 20 µM DM, *n* = 154; 30 µM DM, *n* = 73. (H–K) Analysis of non-canonical BMP pathway. (H) Western blot analysis of animal caps treated with SB203580 (SB20). As expected, activity of p38 (P-p38) is inhibited in caps treated with Noggin and SB20. (I) Canonical signaling through pSmad1/5/8 is not affected by SB20 treatment. (J) SB20 treatment fails to induce the expression of neural genes, *ncam*, *nog*, and *otx2*, compared to DNA histone H4 (h4) loading control; *N* = 3. (K) Animal caps treated with 1 µM SB20 fail to incorporate into host retina, but a few animals have transplanted cells in the brain (*n* = 53). Treatment with 1 µM SB20 and 20 µM DM, compared to 20 µM DM alone, produces the same percentage of host animals with transplanted cells in the retina, while there is a slight increase the number of animals with donor cells in the brain (*n* = 97). Western blots, WB; reverse transcription-PCR, RT-PCR; animal cap transplant assay, ACT assay. Error bars  =  ±s.e.m.; *p<0.05; **p<0.01; ***p<0.001; ns, not significant.

Noggin contributes to neural induction by antagonizing BMP4 (Zimmerman et al., 1996). If BMP inhibition is all that is required for eye formation, we reasoned that blocking this pathway by other means would also generate retinal tissue. BMP receptors signal through two different downstream pathways: the canonical signaling pathway through Smad1/5/8, and the non-canonical pathway through TAK1/TAB and p38 MAPK (Fig. 1A, reviewed by Nohe et al., 2004; Eivers et al., 2008), which are both implicated in the regulation of neural induction (von Bubnoff and Cho, 2001). The small molecule chemical inhibitor dorsomorphin (DM) can selectively inhibit Smad1/5/8 phosphorylation (canonical pathway), leaving p38 phosphorylation (non-canonical pathway) unaffected (Yu et al., 2008; Boergermann et al., 2010). In order to determine which downstream signaling cascade is responsible for retinal specification, pluripotent cells were isolated from the pre-gastrula stage embryo (stage 9), treated with DM and grown to neural plate stage (stage 15; Fig. 1B). As previously reported (Yu et al., 2008), we observed that treatment with dorsomorphin inhibited Smad1/5/8 phosphorylation (pSmad1/5/8; Fig. 1C). While 10 µM DM significantly reduced pSmad1/5/8 (41±8% band density wrt YFP control), treating cells with 30 µM DM (7±2%) repressed pSmad1/5/8 as efficiently as Noggin (3±2%). From these results, we predicted that 30 µM DM would generate eyes from pluripotent cells as efficiently as Noggin treatment.

To test this, we used the Animal Cap Transplant (ACT) assay to determine the fate of the treated cells (Viczian and Zuber, 2010; Fig. 1B). Depending on the cell fate decisions made between isolation and transplantation, donor cells can contribute to the skin, brain, or eye of host embryos. Untreated pluripotent cells differentiate as atypical epidermis in culture or when transplanted to the host eye primordium (Fig. 1D; Woodland and Jones, 1987; Viczian et al., 2009). When treated with Noggin, donor cells differentiated as retinal cells in all host animals (Fig. 1E). Interestingly, donor YFP-expressing pluripotent cells treated with 30 µM DM transformed into retina in only 68±8% of host animals (Fig. 1F,G). We knew that DM was neuralizing the tissue, since we found that DM treatment was able to convert donor cells to brain cells in a similar percentage of animal as donor cells treated with Noggin (Fig. 1G). Increasing the concentration of DM to 40 µM, resulted in cell death. Since inhibition of BMP/Smad signaling could not mimic Noggin's retinal integration efficiency, we hypothesized that inhibition of the non-canonical pathway may fill this gap.

The non-canonical pathway activates p38 MAPK (hereby referred to as p38) via the Tak1-Tab1-XIAP-NRAGE complex (Kendall et al., 2005). Blocking p38 phosphorylation (P-p38) using the small molecule inhibitor SB203580 (SB20) was found to induce expression of the neural gene markers, *ncam*, *nog*, and *otx2* (Goswami et al., 2001). We found that treatment of pluripotent cells with increasing concentrations of SB20 resulted in a dose-dependent repression of P-p38 (Fig. 1H), whereas the canonical pathway (pSmad1/5/8) was unaltered (Fig. 1I). However, we found that *ncam* was only slightly induced at 1 µM SB20, and *noggin* and *otx2* expression was undetectable compared to our Noggin-injected control (Fig. 1J). Consistent with this observation, pluripotent cells treated with 1 µM SB20 failed to form retina and infrequently appeared in host brain tissue (Fig. 1K). With 10 µM or 20 µM SB20 treatment, donor cells remained in the skin, or in the mesenchyme outside the eye (10 µM SB20, *n* = 35; 20 µM SB20, *n* = 28; data not shown). These data suggested to us that inhibiting non-canonical BMP signaling via p38 was not sufficient to induce retinal cell formation. Taken together, we concluded that inhibition of canonical BMP/Smad signaling is sufficient to induce retinal formation, but it cannot mimic the efficiency with which Noggin can transform pluripotent cells into retina.

### High concentrations of Noggin inhibit Activin signaling through Smad2

In addition to binding BMPs, Noggin can also bind Activin ligands; however it was never tested whether this binding event could inhibit the downstream functions of the Activin pathway (Bayramov et al., 2011). Therefore, we investigated Noggin's ability to inhibit Activin signaling though the modulation of Smad2 phosphorylation (pSmad2). We first determined the optimal concentration of Noggin for maximal retinal integration by the ACT assay (supplementary material Fig. S1A–F). We observed that donor cells injected with 20 pg of Noggin generated retina in all host animals (supplementary material Fig. S1A–F). If Noggin acts only on the BMP pathway, then we would expect that pSmad1/5/8 but not pSmad2 would be repressed in these cells. Instead, we found that injecting 20 pg of Noggin reduced the level of pSmad2 to 8±6% of untreated cells (supplementary material Fig. S1G,H). While Noggin clearly affected endogenous Smad2 activity, we observed that Smad2 levels were low in animal cap cells. To determine how low, we measured the transcript and protein levels of Smad2 in animal caps (supplementary material Fig. S1I). We used qRT-PCR to determine that for every 1000 copies of *h4*, there were, on average, 58 copies of *smad1* and 18 copies of *smad2* mRNA, a ratio of about 3. We also performed quantitative western blot analysis and found that Smad1 levels were over 30 times higher than Smad2. Together, these results indicate that when Noggin induces retina formation in animal caps, it reduces endogenous Smad2 activity, which is present at low levels in animal caps.

**Fig. 2.**
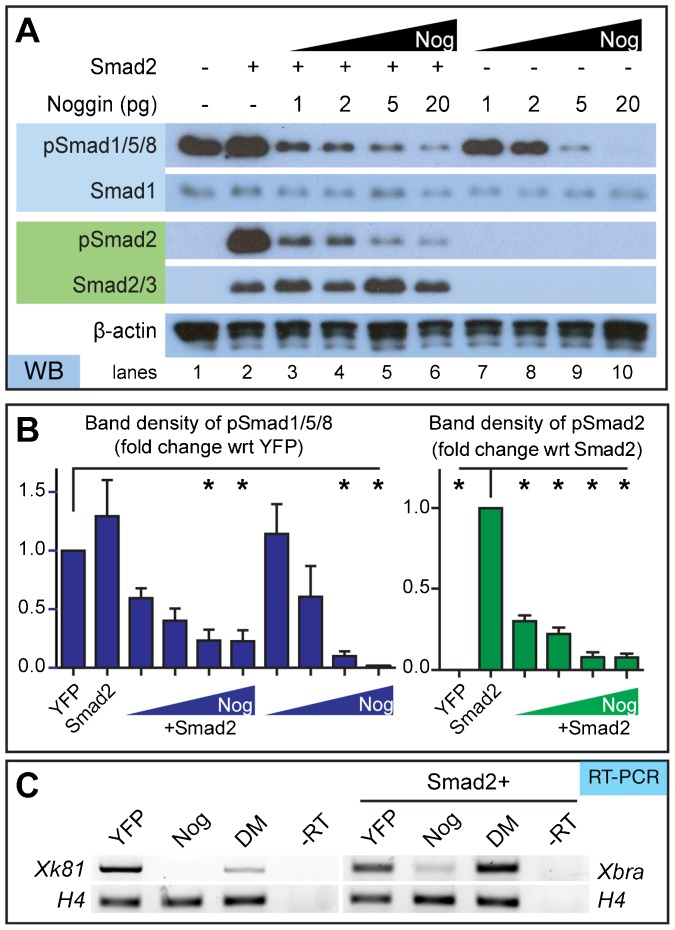
Noggin inhibits Smad1/5/8 and Smad2 phosphorylation in a concentration-dependent manner. (A) Western blot of animal caps isolated from embryos injected with specified amount of Noggin RNA with and without 50 pg Smad2 RNA. Smad1, Smad2, and β-actin served as loading controls. (B) Densitometric analysis of western blots shows that higher concentrations of Noggin inhibit pSmad1/5/8 and pSmad2 (*N* = 3). (C) Noggin inhibits BMP and Activin pathway-specific gene transcription. Noggin-treated caps can inhibit the epithelia marker, *xk81*, and mesoderm marker, *xbra*, as determined through RT-PCR. Conversely, DM affects BMP, not Activin, pathway gene transcripts since it can only affect *xk81* expression; *N* = 3. Error bars  =  ±s.e.m.; *p<0.05.

Activin signaling (via Smad2 phosphorylation) can be stimulated in animal cap cells as early as stage 6 up to stage 11 by adding the Activin ligand (Grimm and Gurdon, 2002). Adding Activin to animal cap cells has allowed closer examination of Smad2 activity in this promiscuous tissue (Chang et al., 1997). Since we were interested in the intrinsic changes regulated by Noggin, we injected a small amount of Smad2 mRNA (50 pg) into embryos, in order to more easily visualize the effect of Noggin on Smad2 activity. While this concentration was high enough to detect pSmad2, it was low enough to prevent animal cap elongation, which is phenotypical of sustained Activin signaling (Asashima et al., 1990; Smith et al., 1990; Thomsen et al., 1990). Consistent with our investigation of endogenous Activin signaling, we found that Noggin could reduce both pSmad1/5/8 and pSmad2 (Fig. 2A). Smad1 and Smad2 protein levels remained constant, suggesting that Smad degradation pathways were not responsible for this change in activity. Although the decrease in Smad2 activity was statistically significant at all Noggin concentrations tested, pSmad2 was repressed more completely when 5 and 20 pg Noggin RNA was injected (Fig. 2B).

If Noggin inhibits inhibits both Smad1/5/8 and Smad2 activity, we expect the downstream gene targets of both pathways to be silenced by Noggin treatment. By conducting RT-PCR analysis, we saw reduction of both the epithelial marker, *xk81*, and mesodermal marker, *xbra*, in the Noggin treated cells (Fig. 2C). On the other hand, DM, which inhibits only BMP signaling, decreased *xk81* expression but not *xbra*. This gives further evidence that Noggin is able to prevent Smad activation and gene transcription of both the BMP and Activin signaling pathways.

### Overexpression of dominant-negative BMP and Activin receptors together mimics Noggin

**Fig. 3.**
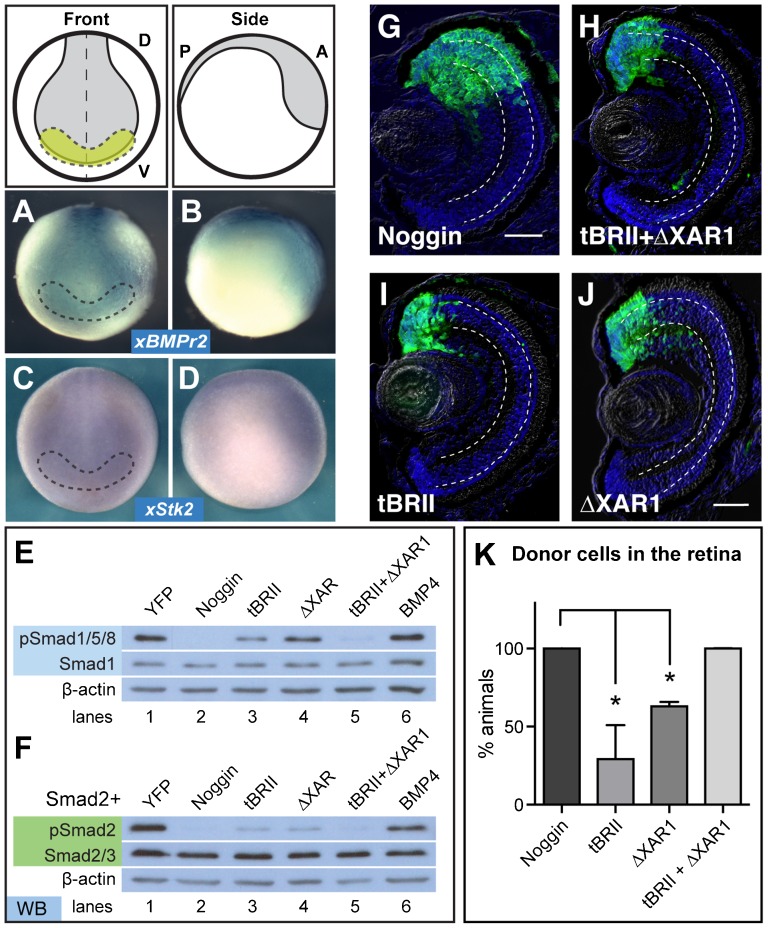
(A-D) Whole mount *in situ* hybridization for BMP (A,B) and Activin (C,D) type II receptors in stage 15 embryos show expression in the eye field (yellow), outlined with the dashed lines. (A) and (C) show the front view, while (B) and (D) show a side view. (E) Expression of truncated BMP (tBRII, 500 pg) or Activin (ΔXAR1, 1 ng) receptors individually suppress pSmad1/5/8, but signaling is repressed further with expression of both tBRII+ΔXAR1. (F) pSmad2 is also repressed with the expression of both tBRII and ΔXAR1. (G-K) Using the ACT assay, the tBRII+ΔXAR1-expressing cells end up in the retina more frequently than either the tBR or ΔXAR1-expressing pluripotent cells. Scale bar, 50 µm. Dashed lines lie on outer and inner plexiform layers. (K) ACT results quantified and statistics determined using a student's t-test, N=2. Error bars = ±s.e.m.; *p,0.05. Green, YFP; blue, DAPI staining. Dorsal ‘D’, ventral ‘V’, posterior ‘P’, anterior ‘A’.

Both BMP4 and Activin signal through specific TGFβ receptors. If Noggin is acting upstream of both pathways, we expect that expression of dominant-negative receptors for both these pathways would mimic Noggin's ability to induce retina formation. We used two mutant membrane receptors, a truncated BMP type II receptor (tBRII) to preferentially block the BMP pathway, and a truncated Activin type II receptor (ΔXAR1) to preferentially block the Activin signaling pathway. Both mutants are able to bind endogenous ligands, but lack their C-terminal domains, preventing the phosphorylation of their complementary type I receptors and downstream signaling Smads (Hemmati-Brivanlou and Melton, 1992; Graff et al., 1994; Frisch and Wright, 1998). To confirm that these pathways have the potential to be activated, we performed *in situ* hybridization on *xBMPrII* (BMP receptor type II) and *xStk2* (Activin A receptor type IIB precursor). We found that both receptors were expressed in the neural plate and eye field of stage 15 embryos (Fig. 3A–D), which is consistent with previous findings (Hemmati-Brivanlou and Melton, 1992). Also consistent with previous findings, we observed a significant decrease in pSmad1/5/8 with injection of tBRII compared to YFP-injected cells (46%; Fig. 3E, lane 3). Similarly, injection of ΔXAR1 significantly decreased pSmad2 (68%; Fig. 3F, lane 4; Hemmati-Brivanlou and Melton, 1992; Frisch and Wright, 1998). We also observed that injection of ΔXAR1 repressed pSmad1/5/8 (68%; Fig. 3E, lane 4), suggesting that ΔXAR1 can disrupt canonical BMP signaling, as others have reported (Wilson and Hemmati-Brivanlou, 1995; Frisch and Wright, 1998). We then wondered if tBRII disrupted Activin signaling. Indeed, we observed that injection of tBRII repressed pSmad2 (15%; Fig. 3F, lane 3) as effectively as injection of ΔXAR1 (17%; Fig. 3F, lane 4), suggesting that tBRII also represses both BMP and Activin signaling. When both are added together, we saw further reduction of both pSmad1/5/8 (12%) and pSmad2 (8%; lanes 5 in Fig. 3E,F), similar to levels observed when injecting cells with Noggin RNA (0%, pSmad1/5/8; 6%, pSmad2; lane 2 in Fig. 3E,F).

If Noggin is blocking both pathways to drive retinal cell fate, then transplanting animal caps expressing both these dominant-negative receptors should mimic the cell fate decisions seen with the Noggin donor cells. This is indeed what we observed. YFP+ donor cells expressing both tBRII and ΔXAR1 (100%; *n* = 43, Fig. 3H) transformed into retina as efficiently as cells expressing Noggin (100%; *n* = 64, Fig. 3G). Expressed individually, neither tBRII (29±12%, *n* = 66, Fig. 3I) nor ΔXAR1 (63±2%, *n* = 40, Fig. 3J) expression transformed the donor cells as efficiently (Fig. 3K); statistically, there was no difference in the efficiency of generating retina between these two samples. These results give evidence to our hypothesis that repression of both Smad1 and Smad2 signaling increases the efficiency of generating retina from pluripotent cells.

### Overexpression of dominant-negative Smad1 and Smad2 construct expands the eye field *in vivo*

**Fig. 4.**
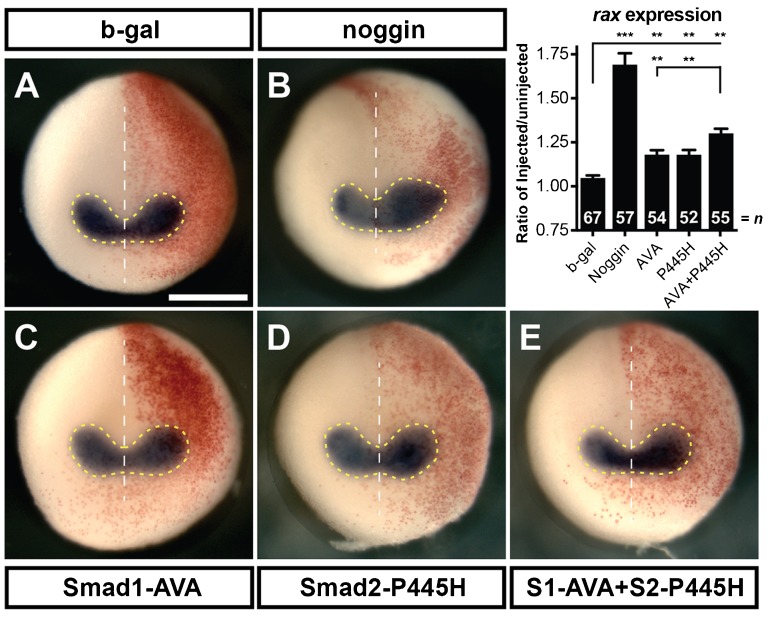
Injection of Smad1-AVA (S1-AVA) and Smad2-P445H (S2-P445H) act additively to cause expansion of eye field. Whole mount *in situ* hybridization for the eye field marker, *rax* (A–E) conducted on stage 15 embryos unilaterally injected with 125 pg of S1-AVA RNA, 3 ng of S2-P445H and 100 pg of β-gal. Area of *rax* expression was calculated by measuring the region within the dashed yellow lines on each side of the midline (white dotted line) as shown. Graph shows the ratio of the area of the injected side to the uninjected side. Red β-gal stain indicates injected side. Scale bar, 500 µm. Error bars  =  ±s.e.m.; **p<0.01; ***p<0.001.

To better understand the role of BMP and Activin inhibition on retinal development *in vivo*, we attempted to block these pathways in whole embryos using two previously characterized dominant-negative Smad mutants. To block BMP signals, we injected a dominant-negative Smad1 mutant (Smad1-AVA) in which the C-terminal phosphorylated serines (Ser-463/465) were mutated to alanines to prevent phosphorylation but maintain receptor and co-factor interactions (Nojima et al., 2010). To block Activin signals, we injected a dominant-negative Smad2 mutant (Smad2-P445H), which harbors a point mutation in the MH2 domain that alters the structure and prevents C-terminal phosphorylation (Eppert et al., 1996). Mutants were unilaterally injected with RNA, including β-gal RNA as a tracer. Neural plate expansion was measured by *in situ* hybridization for the eye field marker, *rax*. The *rax* expression pattern remained bilaterally symmetric with injection of β-gal (Fig. 4A; injected:uninjected ratio of 1.05±0.01, *n* = 67). If BMP and Activin repression are both necessary for efficient eye formation, we would expect to see an expansion of the eye field when both mutants are expressed. Indeed, we saw that when injected alone, Smad1-AVA and Smad2-P445H slightly expanded *rax* expression domains (Fig. 4C; 1.18±0.02, *n* = 54; Fig. 4D; 1.18±0.03, *n* = 52, respectively). Eye field expansion was further increased by the injection of both Smad1-AVA and Smad2-P445H together (Fig. 4E; 1.30±0.03, *n* = 54). This suggests that, in the embryo, reducing Smad2 activity enhances the effect of reducing Smad1 activity to expand *rax* expression. However, this expansion was not as great as injecting Noggin alone (Fig. 4B; 1.69±0.07, *n* = 57). It is known that these constructs fail to completely abolish endogenous Smad1 or Smad2 activity (Nojima et al., 2010; Eppert et al., 1996; Hoodless et al., 1999; Prunier et al., 2001). This would explain the difference we observed between *rax* expansion in Noggin samples versus those treated with the dominant-negative Smads.

### Co-inhibition of BMP and Activin signaling using chemical inhibitors drives pluripotent cells to a retinal cell fate

**Fig. 5.**
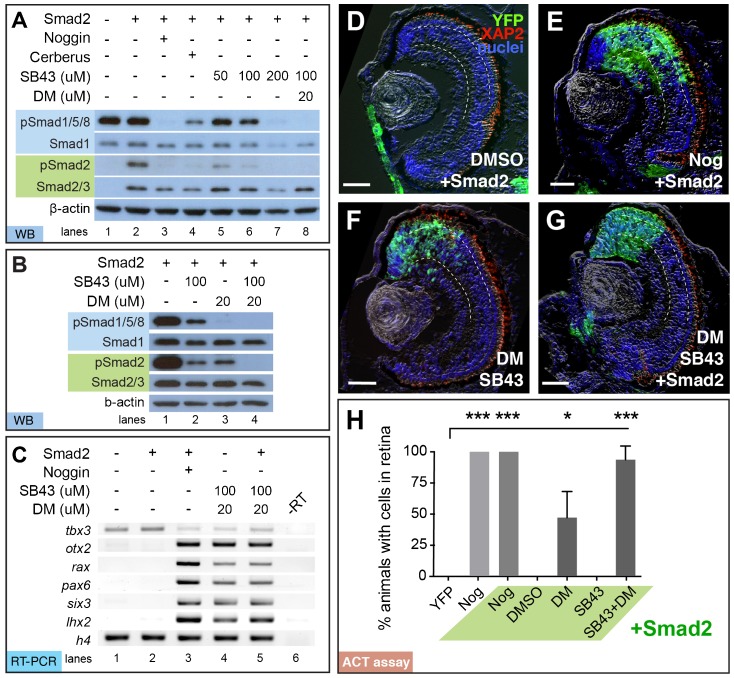
Dual inhibition of Smad1/5/8 and Smad2 activity with chemical inhibitors DM and SB43 is sufficient to drive retinal formation. Embryos were injected with Smad2 (50 pg), Noggin (20 pg), or Cerberus (1.6 ng) RNA and then treated with 50, 100, or 200 µM of SB43 and/or 20 µM DM. (A,B) Treatment with 100 µM SB43 + 20 µM DM (SB43+DM) mimics Noggin's ability to suppress pSmad1/5/8 and pSmad2, as determined by western blot. (B) Suppression of both pSmad1/5/8 and pSmad2 is only complete with treatment of both SB43+DM. Smad1, Smad2, and β-actin served as loading controls. (C) RT-PCR analysis of animal caps shows that Noggin or SB43+DM±Smad2 induces expression of *rax*, *pax6*, *six3*, and *otx2*, while repressing *tbx3*. Histone H4 (*h4*) was used as a loading control. (D–H) DM+SB43±Smad2 treatment mimics the retinal integration efficiency of Noggin. (D) Smad2-injected cells treated with DMSO remain in the skin, while (E) Nog+Smad2 injected cells form retina in all animals. (F) SB43+DM treatment alone and (G) with Smad2 direct pluripotent cells to the retina. Scale bar, 50 µm. Dashed lines lie on outer and inner plexiform layers. (H) Quantification of ACT assay results shows the synergistic effect of adding DM and SB43 to generate retina (*N* = 3). Green, YFP; red, rod photoreceptor marker, XAP2; blue, DAPI staining. Error bars  =  ±s.e.m.; **p<0.01; ***p<0.001.

If complete inhibition of both pathways is required for efficient retina formation in pluripotent cells, we would expect blocking both Smad1 and 2 phosphorylation by chemical inhibitors would produce retinal cells in the pluripotent tissue. Again, DM was used to inhibit pSmad1/5/8, while the small molecule inhibitor, SB431542 (SB43), was used to inhibit pSmad2 (Inman et al., 2002; Laping et al., 2002). We first determined the optimal concentration of each chemical inhibitor to selectively inhibit each pathway. First, treating animal caps with SB43 significantly repressed pSmad2, with only a slight reduction of pSmad1/5/8 at 50 µM (99.5±15.3%; Fig. 5A, lane 5) and 100 µM (75.3±25.4%; Fig. 5A, lane 6), whereas treatment with 200 µM SB43 significantly reduced pSmad1/5/8 (39±27%; Fig. 5A, lane 7). Consistent with previously published data, we found that 30 µM lowered Smad2 activity, so we used dorsomorphin at 20 µM, (Fig. 5B, lane 3; Yu et al., 2008). Treatment with 100 µM SB43 and 20 µM DM (SB43+DM) was sufficient to repress both pSmad1/5/8 and pSmad2 (3.0±1.7%, 9.7±1.3%, respectively; Fig. 5A, lane 8) as effectively as injection with Noggin (2.7±2.2%, 7.0±4.0%; Fig. 5A, compare lanes 3,8).

Noggin has been shown to induce the EFTFs and anterior neural marker, *otx2* (Zuber et al., 2003). If SB43+DM treatment is sufficient to mimic Noggin's ability to generate retina, then we would expect that SB43+DM treatment is sufficient to increase EFTF expression in animal caps. Gene expression was determined by conducting semi-quantitative RT-PCR. Consistent with our previous findings, *tbx3* was the only EFTF detected in untreated caps (Fig. 5C, lane 1). As expected, injection of Smad2 failed to induce EFTF expression (Fig. 5C, lane 2), whereas injection of 20 pg Noggin was sufficient to induce the expression of *otx2*, *rax*, *pax6*, *six3*, and *lhx2*, and repress *tbx3* (Fig. 5C, lanes 3). SB43+DM treatment was sufficient to induce these same transcripts with and without injection of Smad2 (Fig. 5C, lanes 4,5). Expression levels of *six3, otx2, and tbx3* were not significantly different in animal caps treated with SB43+DM versus those injected with Noggin RNA, wherea*s rax, pax6, and lhx2* expression was about half (supplementary material Fig. S2).

Finally, to determine if the SB43+DM treatment is sufficient to direct a retinal cell fate, we again performed the ACT assay. Consistent with previous findings, 20 µM DM-treated animal caps formed retina in only 47% of animals, while tissue treated with YFP or Smad2 did not contribute to eye formation (Fig. 5D,H). Treatment with 100 µM SB43 alone failed to form retina, suggesting that inhibition of Activin signaling was not sufficient to induce retina alone. However, YFP+ animal caps treated with both SB43 and DM transformed into retinal cells in all animals (100%; *n* = 22; Fig. 5F). Again, the presence of Smad2 with Noggin RNA (100%; *n* = 65; Fig. 5E) or Smad2 with SB43+DM caps (94±6%; *n* = 55; Fig. 5G) showed no impact on retinal integration efficiency. Thus, SB43+DM treatment mimics Noggin's retinal formation efficiency. All of these results support our hypothesis that inhibition of both Activin and BMP pathways together allows the generation of retina as efficiently as Noggin (Fig. 5H).

### Chemical inhibitors SB431542 and dorsomorphin expand *rax* expression

**Fig. 6.**
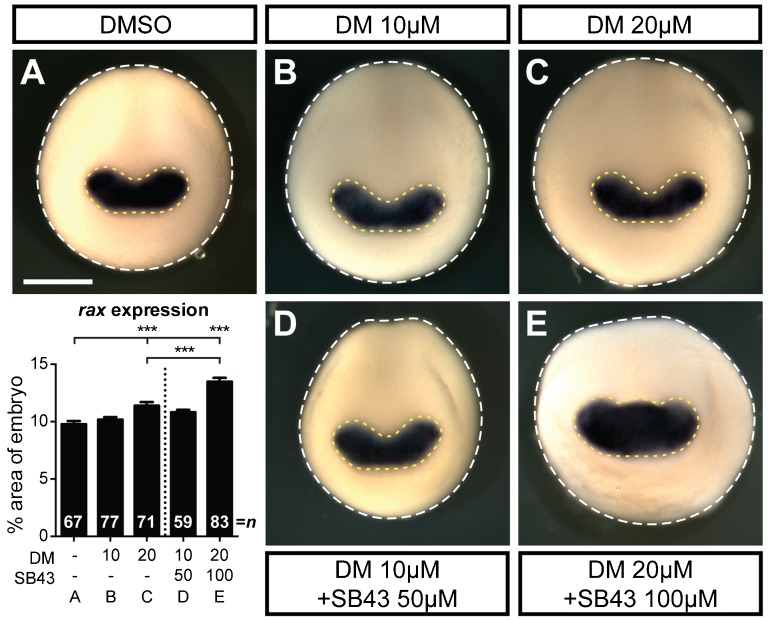
DM and SB43 treatment results in eye field expansion. (A–E) *In situ* hybridization for the eye field marker, *rax*, in embryos treated with DMSO (A), DM (B,C) or DM+SB43 (D,E) from stage 9–15. Eye field expansion was determined by measuring the area of *rax* expression (dashed yellow line) normalized to the area of the dorsal face of the embryo (dashed white line), as shown in the graph. Error bars  =  ±s.e.m.; ***p<0.001. Dotted line on graph separates single treatment from dual treatment. Scale bar, 500 µm; n = number of embryos.

The above results suggest that BMP and Activin inhibition is sufficient to direct isolated pluripotent cells to generate retina. We were next interested if the same phenomenon was true *in vivo*. We treated embryos with varying concentrations of DM and/or SB43, and probed for rax expression by whole mount *in situ* hybridization. We hypothesized that if treatment with DM and SB43 is sufficient to drive retina formation *in vivo*, then the *rax* expression domain would be expanded. We observed that embryos treated with 10 µM DM resulted in an insignificant expansion of the eye field compared to DMSO treated embryos (Fig. 6A,B; DMSO, 9.8±0.3% of the anterior embryo face; DM 10 µM, 10.8±0.2%). We first see expansion of the *rax* expression domains with treatment of 20 µM DM (Fig. 6C, 11.4±0.3%). This expansion was further increased by treatment with both 20 µM DM and 100 µM SB43, as previously used in our transplant assays (Fig. 6E, 13.5±0.3%). Treatment with a more dilute SB43+DM treatment (10 µM DM and 50 µM SB43) resulted in no significant expansion (Fig. 6D, 10.8±0.2%). Thus, inhibiting BMP and Activin signaling using these two chemical inhibitors can also promote eye field expansion *in vivo*.

### Cerberus, but not Follistatin, is able to generate retinal tissue at the same efficiency as Noggin

**Fig. 7.**
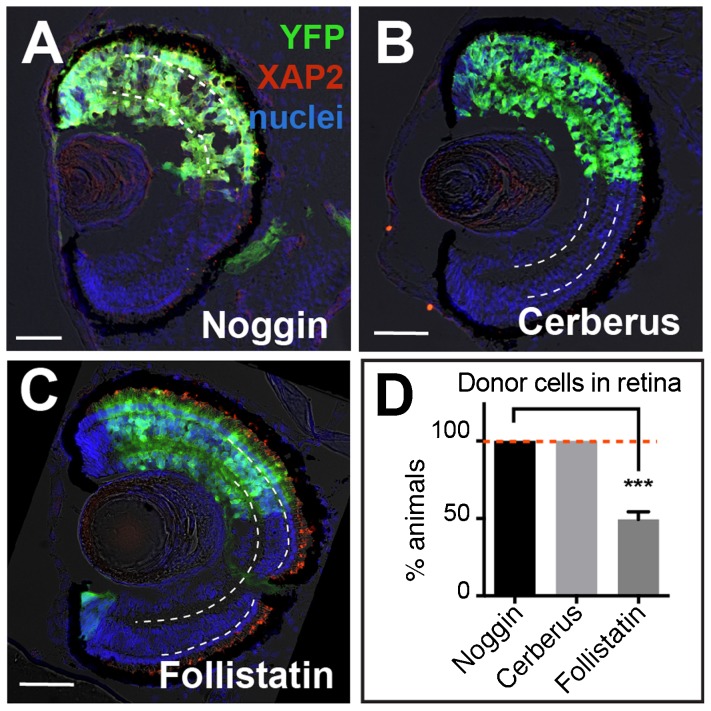
Follistatin is less efficient than Cerberus or Noggin at specifying retina. (A–C) YFP+ donor cells expressing Noggin, Cerberus, and Follistatin all contribute to the retina. However, Noggin- and Cerberus-treated cells formed retina in all animals while cells expressing Follistatin had a significantly lower retinal integration efficiency (D). On retinal sections, dashed white lines lie on outer and inner plexiform layers. Green, YFP donor cells; red, rod photoreceptor marker XAP2; blue, DAPI staining. YFP, 500 pg, *n* = 29; Nog, 20 pg, *n* = 65; Cerberus, 1.6 ng, *n* = 77; Follistatin, 1200 pg, *n* = 53; *N* = 3. Scale bars, 50 µm. Error bars  =  ±s.e.m.; ***p<0.001. Dashed red line on the graph marks Noggin/Cerberus treatment, highlighting the difference from Follistatin treatment.

If blocking both the BMP and Activin pathways are necessary to transform pluripotent tissue into retina, then adding a known antagonist of both pathways, like Cerberus (Piccolo et al., 1999), should generate retinal tissue as efficiently as Noggin. Using the ACT assay, we observed Cerberus-expressing pluripotent cells formed retina with a similar frequency as Noggin (Cerberus, 98±2%, *n* = 77; Noggin, 100%, *n* = 65, Fig. 7A,B). Conversely, if we used an antagonist that favored blocking the Activin pathway over the BMP pathway, like Follistatin (Thompson et al., 2005), we hypothesized that less of these treated animal caps to become eye tissue. Animal caps were isolated from embryos injected with an increasing concentration of Follistatin RNA (400 pg, 800 pg, 1200 pg, and 1600 pg) and the retinal integration efficiency was tested by ACT assay (supplementary material Fig. S3). Animal caps injected with 1600 pg did not survive, but those injected with lower concentrations formed retina in 21±10%, 41±11%, and 50±3% of animals, respectively. To be sure that the exogenous Follistatin can still function as a neural inducer, we scored the same sections for brain formation based on morphology and cellular location. The percentage of animals that contained transplanted cells found in the brain were comparable between Noggin- (80±6%; *n* = 80) and Follistatin-treated (74±10%; *n* = 60) cells. Thus, factors that repressed both BMP and Activin signals, such as Noggin and Cerberus, were more efficient retinal-inducers than Follistatin, an Activin-biased antagonist.

## Discussion

**Fig. 8.**
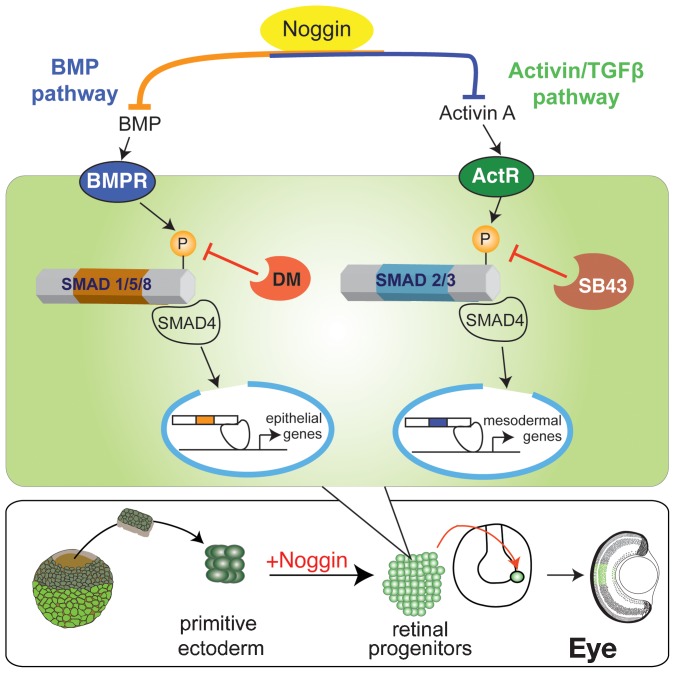
Model of the intracellular pathways altered by Noggin to specify retinal progenitors. DM and SB43 inhibit activation of Smads by BMP and Activin receptors, respectively. In both pathways, gene transcription fails to drive epithelial or mesoderm specifying genes, which allows primitive ectoderm to take on a retinal progenitor cell fate.

We have found that Noggin can direct pluripotent *Xenopus* animal caps to form fully functional retina (Viczian et al., 2009). Others have shown that Noggin overexpression alone is sufficient to generate retina in ventral blastomere cells normally, not fated to become retina (Moore and Moody, 1999). However, the molecular mechanism between Noggin treatment and retinal specification is still unknown. Our studies begin to address this question by investigating the signaling cascades downstream of Noggin to better understand how retina is specified. We present results in support of Noggin acting as both a BMP and Activin antagonist to regulate the specification of retinal progenitors (Fig. 8). We further investigated the role of each pathway by inhibiting their activity in pluripotent animal caps by expressing dominant-negative type II receptors tBRII and ΔXAR1, or by treating with the small molecule chemical inhibitors, dorsomorphin and SB431542. In each case, our results suggest that inhibition of BMP or Activin signaling alone led to mediocre retinal specification. However, when signaling through both pathways was inhibited, the treated cells expressed the EFTFs and generated retinal cells, as efficiently as Noggin. This suggests that at high concentrations Noggin induces retina by modulating both pathways. Cerberus, another BMP antagonist that is known to inhibit both BMP and Activin pathways, was able to specify retina as efficiently as Noggin. Follistatin, which is known to predominantly inhibit Activin signaling, was not as efficient. Collectively our results suggest that the dual inhibition of the BMP and Activin pathways directs pluripotent animal caps to generate retina as efficiently as Noggin.

Although it was previously shown that inhibition of the BMP signaling pathway alone could drive neural induction in pluripotent *Xenopus* cells, the current model of efficient neural induction requires the inhibition of both BMP and Activin signaling via the suppression of Smad1 and Smad2 signaling (Chang and Harland, 2007). This was discovered by blocking TGFβ signaling in ventral blastomeres, which lead to neural induction in the absence of mesoderm. Overexpressing low levels of the dominant-negative BMP receptor type IA, Chang and Harland showed increased efficiency of neural induction when adding dominant-negative Activin receptor type IB. When a high dose of this BMP inhibitor was used, the effect of the dominant-negative Activin receptor was lost (Chang and Harland, 2007). This illustrates how low levels of Smad2 activity can reduce efficient neural induction, just like we observed in this study for efficient retina formation. We used the dominant-negative type II receptors or dominant-negative R-Smads to favor Activin repression in the presence of low levels of BMP repression in order to efficiently induce retina formation. We found that when we used higher concentrations the dominant-negative BMP receptor or the dominant-negative Smad1 construct, we mask the additive effect of Activin inhibition (data not shown). This is not that surprising as we found that injection of 500 pg tBrII in animal cap cells caused a reduction of pSmad2 similar to injection of ΔXAR1. Others have used twice as much tBr RNA in their overexpression studies, before it was possible to check for Activin pathway repression (Frisch and Wright, 1998; Suzuki et al., 1997). Since we found that Smad2 is present in much lower levels than Smad1 at this developmental time point, we would expect that both pathways would be inhibited when using unnaturally high concentrations of the dominant-negative BMP pathway components.

Both BMPrII and XAR1 (also known as xActRIIB) can act promiscuously to bind the ligands and type I receptors of the opposite pathways due to the high degree of sequence homology shared in protein interaction domains (Chang et al., 1997; Rejon et al., 2013). Analysis of the binding affinities for cross-pathway ligand-receptor interactions showed that there is less than a 10-fold difference in binding affinities for ligands to the receptors of the opposite pathway (Heinecke et al., 2009). Because of this, we were not surprised that the truncated versions of these receptors also exhibit the same cross-talk. Here, we reported that ΔXAR1 and tBRII reduced both Smad1/5/8 and Smad2 phosphorylation when injected individually. Although such promiscuity has been suggested for ΔXAR1 (Frisch and Wright, 1998), we were surprised to see such a high effect of tBRII on Smad2 phosphorylation. As we stated above, it is likely that these off-target effects have previously been overlooked because of the inability to detect Smad activity directly. Taken together, this points to the truncated receptors not being efficient methods of inhibiting pathways specifically.

Endogenous Smad2 activity was present in animal cap cells, but at low levels. However, we found that all of the Activin signaling components are present and primed for activity because when we injected small amounts of Smad2 RNA, cells stimulated Smad2 phosphorylation, as visualized by western blot. Analysis of Smad1 and Smad2 protein versus transcript levels revealed that the levels of Smad1 versus Smad2 protein are 10-fold lower than *smad1* versus *smad2* mRNAs. This could simply be due to the antibodies having different binding affinities for their respective antigens. Alternatively, it is possible that the translation of Smad1 and Smad2 is differentially regulated, or Smad2 is degraded more efficiently than Smad1. Since the endogenous activity of the BMP and Activin signaling pathways are so closely regulated throughout development, future experiments could focus on these potential secondary mechanisms of activity modulation.

Studies on human embryonic stem cells have also observed that the dual inhibition of BMP and Activin signaling caused neural induction. When cells were exposed to BMP- and Activin-inhibiting agents such as the chemical inhibitor, SB431542, or the antagonists Lefty, Cerberus, or Noggin, the cells aggregated into neural rosettes and expressed neural markers in greater than 80% of all cells treated (Smith et al., 2008; Chambers et al., 2009). Consistent with this model, we observed that retinal specification was most efficient when pluripotent cells were treated with reagents that blocked both pathways. Future studies could determine if using these reagents could generate even more retinal cells in pluripotent stem cell cultures.

We also discovered that blocking the non-canonical BMP4/TAK1 pathway, via a P-p38 inhibitor, SB203580, has a limited effect on neural induction and no effect on retinal generation. It was believed that inhibition of p38 was sufficient to induce expression of *ncam*, *nog*, and *otx2* in stage 12 animal caps (Goswami et al., 2001; Liu et al., 2012). However, our analysis showed that this induction was suppressed by stage 15 animal caps, which raises the possibility that later signals override neural induction by p38 inhibition. Furthermore, p38 inhibition by SB203580, or in the presence of dorsomorphin, failed to enhance retinal specification in our ACT assay. We found a small number of animals with transplanted cells in the brain but none in the retina. These results suggest that p38 has a negligible effect on retinal or neural formation, but future studies could test whether other members of the BMP4/TAK1 pathway may play a role in retinal specification.

Inhibition of both BMP and Activin signaling with the use of chemical inhibitors or dominant-negative Smads expanded the eye field marker, *rax*, *in vivo*. Interestingly, the *rax* expression domain is still limited, suggesting that anti-retinal or anti-neural signals may be present in the posterior embryo. One potential signal is the Wnt/β-catenin signaling cascade, which morphogenically regulates anteroposterior neural patterning (Kiecker and Niehrs, 2001; Niehrs et al., 2001). The Wnt inhibitor Dickkopf-1 has been used in coordination with Noggin and insulin-like growth factor-1 to generate retinal progenitors from human embryonic stem cells (Lamba et al., 2006; Lamba et al., 2009). Furthermore, both the BMP and Activin pathways have been shown to interact with the canonical Wnt pathway during development, by direct protein-protein interactions and by transcriptional regulation (reviewed in Guo and Wang, 2009). Smad1 phosphorylation is stabilized by Wnt signaling (Fuentealba et al., 2007), and activated Smad1, Smad2, and Smad3 have been shown to complex with β-catenin to regulate gene transcription (Labbé et al., 2000). Therefore, it is possible that inhibition of both BMP and Activin signals could have a secondary effect on Wnt signaling, however the significance of this effect remains unknown. Inhibition of all R-Smad signaling could then prevent nuclear trafficking and the lack of β-catenin/Smad complexes may quench Wnt signaling. Alternatively, work in Drosophila also suggests that inactivated Smad1/5/8 can act as cofactors to activate Wnt pathway genes (Eivers et al., 2009; Eivers et al., 2011). Therefore, further investigation into the activity of these pathways would be required to gain a better understanding of how these pathways interact during retinal specification.

## Supplementary Material

Supplementary Material
