# Supplementary material for: Efficient retina formation requires suppression of both Activin and BMP signaling pathways in pluripotent cells
Source: Biol Open. 2015 Mar 6;4(4):573–83. doi: 10.1242/bio.20149977 (PMC4400599; doi:10.1242/bio.20149977)
Supplement: Supplementary Material [file supp_4_4_573__index.html]

Efficient retina formation requires suppression of both Activin and BMP signaling pathways in pluripotent cells — Efficient retina formation requires suppression of both Activin and BMP signaling pathways in pluripotent cells — Supplementary Material 

# Efficient retina formation requires suppression of both Activin and BMP signaling pathways in pluripotent cells

## bio.20149977 Supplementary Material

**Files in this Data Supplement:**

- Supplementary Material - Kimberly A. Wong et al. doi: 10.1242/bio.20149977
