# Supplementary material for: Efficient retina formation requires suppression of both Activin and BMP signaling pathways in pluripotent cells
Source: Biol Open. 2015 Mar 6;4(4):573–83. doi: 10.1242/bio.20149977 (PMC4400599; doi:10.1242/bio.20149977)
Supplement: Supplementary Material [file supp_bio.20149977_bio.20149977-s1.pdf]

Biology Open (2015) 000, 1–11 doi:10.1242/bio.20149977

Supplementary Material
Kimberly A. Wong et al. doi: 10.1242/bio.20149977

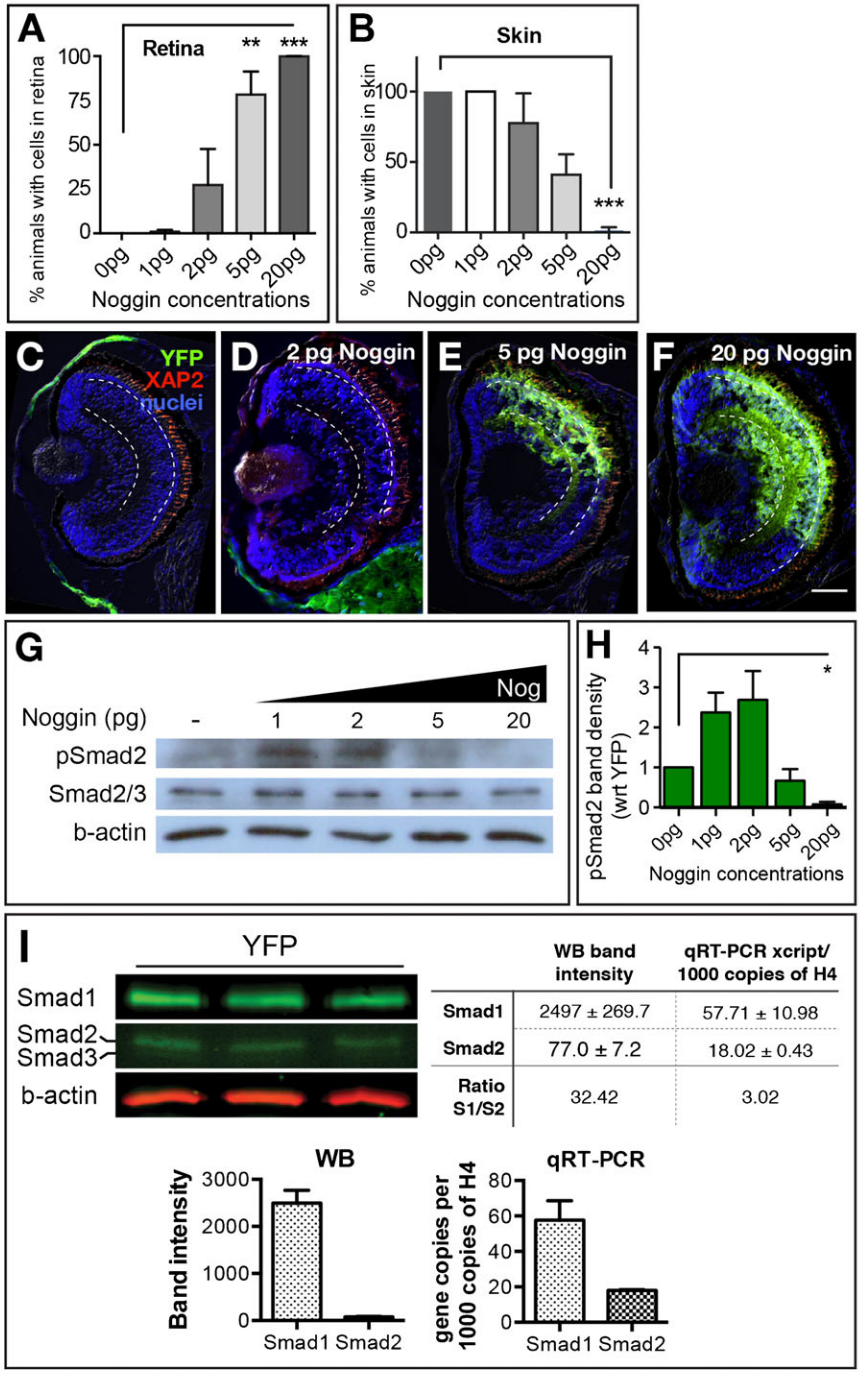

Fig. S1. Effect of increasing concentration of Noggin increases efficiency of retina formation while blocking Activin pathway signaling. (A–F) The ACT assay was conducted with donor tissue from embryos injected with increasing concentrations of Noggin RNA. (A) Animal cap cells injected with 5 and 20 pg of Noggin (and 500 pg YFP RNA as a tracer) form retina in nearly all animals (75 and 100%).
(B) Conversely, the percentage of animals with transplanted YFP+ cells forming skin decreases with higher concentration of Noggin. (C–F) Representative sections from each experimental condition. Green, YFP; red, rod photoreceptor marker, XAP2; blue, DAPI staining. Noggin titer: YFP, *n*=60; 1 pg, *n*=65; 2 pg, *n*=63; 5 pg, *n*=65; 20 pg, *n*=61; *N*=3. Scale bar, 50 μM. (G,H) Western blot analysis of endogenous pSmad2 activity in animal caps injected with 0 to 20 pg of Noggin RNA. Low Noggin concentrations (1–2 pg) results in a slight boost in signal, while 5 and 20 pg represses pSmad2 (*N*=3). (I) Smad2 protein and mRNA is present in untreated animal caps, but at a lower concentration than Smad1. Quantitative fluorescent western blot analysis for Smad1 and Smad2/3 in animal caps injected with YFP, normalized to β-actin control. All three biological replicates are shown. Transcript was extracted from untreated animal caps for qRT-PCR analysis. Absolute quantification of *smad1* and *smad2* mRNA transcripts (xcripts) are represented 'per 1000 copies of H4 gene'. This was measured by standard curve method, using a known quantity of a sequenced plasmid template. All analysis conducted on each sample twice in three biological replicates.

Biology Open (2015) 000, 1–11 doi:10.1242/bio.20149977

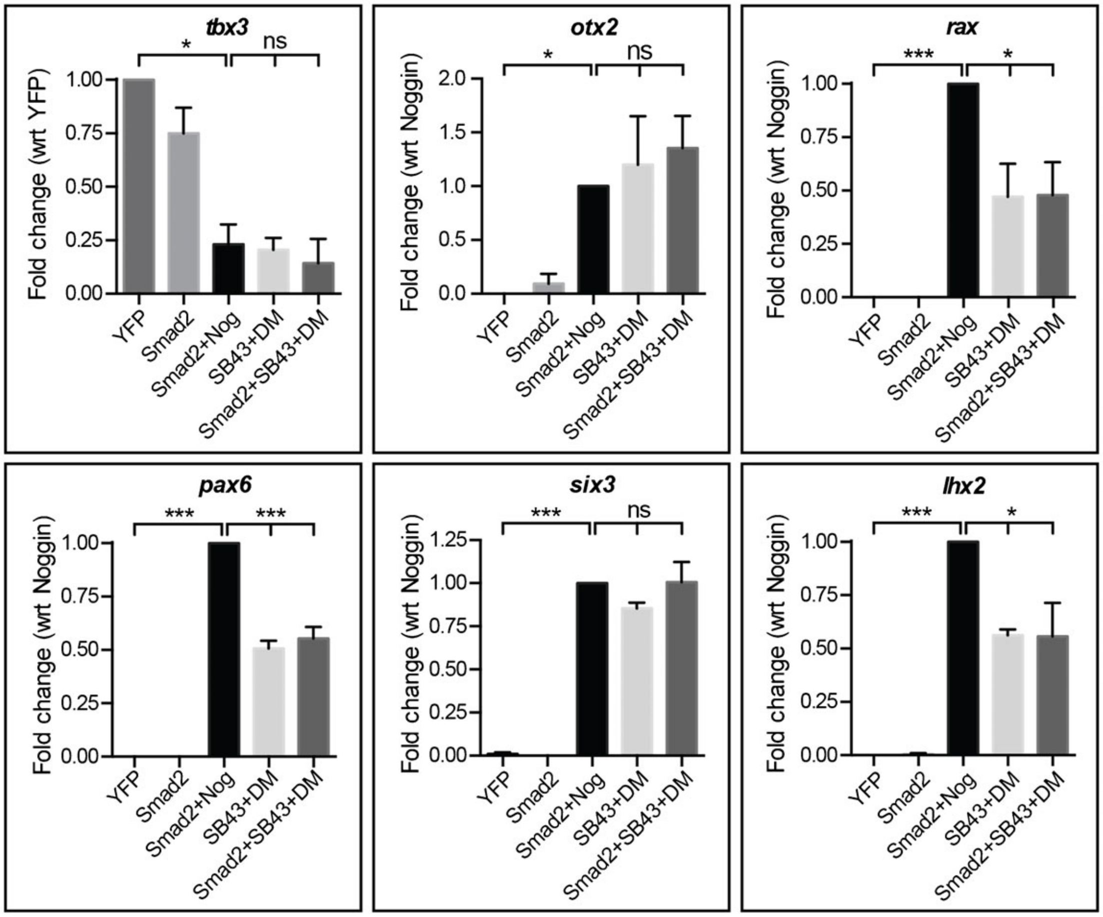

Fig. S2. Reproducible expression of EFTF by treatment with Noggin or SB43+DM. Densitometric analysis of ChemiDoc gel images (BioRad) of PCR reactions for the EFTFs shown in Fig. 5 using primer pairs listed in supplementary material Table S3. Samples were normalized to histone H4. Biological replicates: *tbx3*, *N*=3; *otx2*, *N*=3; *rax*, *N*=3; *pax6*, *N*=3; *six3*, *N*=3; *lhx2*, *N*=2.

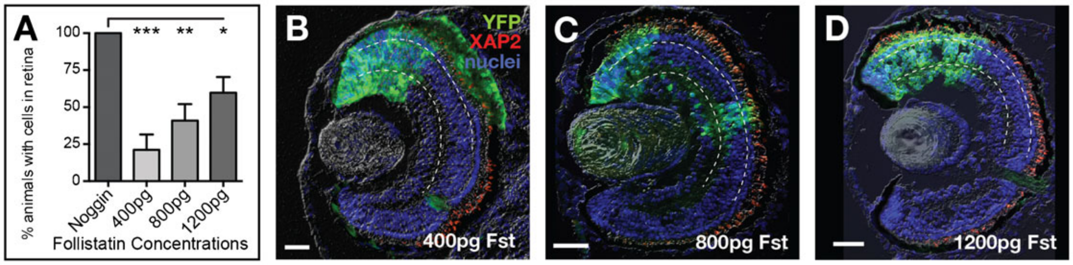

Fig. S3. Effect of increasing concentration of Follistatin in ACT assay. (A) Animal caps were injected with 400, 800, or 1200 pg of Follistatin RNA with YFP RNA. Increasing the amount of injected Follistatin RNA increased the ratio of animals showing successful retinal integration from approximately 20% to 60%. However, compared to Noggin (*n*=80), there were still significantly fewer animals with transplanted cells in the retina. Higher concentrations of Follistatin caused cell death in animal caps. (B–D) Representative sections from each experimental condition. Follistatin titer: 400 pg, *n*=79; 800 pg, *n*=84; 1200 pg, *n*=60; *N*=3. Scale bars, 50 µm. Error bars= ±s.e.m.; *p<0.05; **p<0.01; ***p<0.001.

Biology Open (2015) 000, 1–11 doi:10.1242/bio.20149977

Table S1. Antibodies used for western blots and IHC

Antibody	Source	Concentration	Catalog number
Mouse anti-XAP2	Developmental Studies Hybridoma Bank	1:25	Clone 5B9
Rabbit anti-GFP	Molecular Probes (Invitrogen)	1:1000	A11122
DAPI (49,6-Diamidino-2-phenyindole, dilactate)	Sigma	10 mg/ml	D9564
Rabbit anti-phospho-Smad1/5/8 (C-term)	Cell Signaling Technology	1:1000	9511
Rabbit anti-Smad1	Cell Signaling Technology	1:1000–2000	9743
Rabbit anti-phospho-Smad2	Cell Signaling Technology	1:500	3108
Rabbit anti-Smad2/3	Cell Signaling Technology	1:1000–2000	5678
Rabbit anti-Phospho-p38 MAPK (Thr180/Tyr182)	Cell Signaling Technology	1:500	9211
Rabbit anti-p38 MAPK	Cell Signaling Technology	1:500	9212
Rabbit anti-β-actin	Cell Signaling Technology	1:2000	4967
Goat anti-rabbit IgG HRP	Fisher	1:5000	PI-31460
Alexa 488 donkey anti-rabbit IgG	Mollecular Probes (Invitrogen)	1:1000	A21206
Alexa 594 goat anti-mouse IgG3	Mollecular Probes (Invitrogen)	1:1000	A21155
Mouse anti-beta-actin	Li-Cor	1:4000	926-42212
IR Dye 800CW goat anti-rabbit	Li-Cor	1:15,000	827-08365
IR Dye 680RD goat anti-mouse	Li-Cor	1:15,000	926-68170

Table S2. Constructs used to generate RNA for experiments or for qPCR standards

Construct	Linearized with	Reference
XBmp4-b+pCS2+	PspOMI	This study
XCerberus+pCS2+	PspOMI	EXRC; XB-CLONE-8773700
XFollistatin+pSP64T	XbaI	EXRC; XB-CLONE-12442174
Dominant-negative BMP receptor type II (tBRII) +pCS2	NotI	(Frisch and Wright, 1998)
Dominant-negative activin receptor type II (ΔXAR1)+pSP64T	EcoRI	Addgene catalog number 17005; (Hemmati-Brivanlou and Melton, 1992)
mSmad1-AVA+pCS2+	NotI	(Nojima et al., 2010)
XSmad2+pCS2R	NotI	This study
XSmad2-P445H+pCS2R	NotI	This study, (Eppert et al., 1996)
XNoggin+pCS2+	NotI	(Smith and Harland, 1992)
VenusEYFP+pCS2+	NotI	(Nagai et al., 2002)
xH4+pGEMTez		This study
xSmad1_frag+pGEMTez		This study
xSmad2_frag+pGEMTez		This study

Table S3. RT-PCR primers

Target	Upstream primer (5′–3′)	Downstream primer (5′–3′)	Reference	Cycles
pax6	GCAACCTGGCGAGCGATAAGC	CCTGGCGTCTCTGGTTCGTA	(Zuber et al., 2003)	30
tbx3	CCTATCCTTGACTTGCTACA	GTTTTGGGGAAGGAGGGTAT	(Zuber et al., 2003)	27
rax	CCCCAACAGGAGCATTTAGAAGAC	AGGGCACTCATGGCAGAAGGTT	(Zuber et al., 2003)	28
six3	TTGTCTGTCTGTCTCTTGTT	TTCTGTGTTTGGTTTATCTC	(Zuber et al., 2003)	33
lhx2	CCGGAGATGCTTTTCCACAG	GTAAGCTCCGACTCCAGGTT	This study	30
otx2	CTGTCCAAGCTCACATACTAACA	CAGAGGTAGTCAGGCTGAGC	This study	40
ncam	CACAGTTCCACCAAATGC	GGAATCAAGCGGTACAGA	(Zuber et al., 2003)	37
noggin	CCAGACCTTCTGTCCTGT	AGTCCAAGAGTCTCAGCA	(Zuber et al., 2003)	43
xbra	CAGTTCATAGCAGTGACCGC	GGCGAACATAATGACCCACC	This study	25
xk81	TCTCGCTTCCTACCTGGAGA	CCATTTCCAGCCTGGTCTTA	This study	26
h4	CGGGATAACATTCAGGGTATCACT	ATCCATGGCGGTAACTGTCTTCCT	(Zuber et al., 2003)	26
xsmad1	TCCACCATGGTTTTCATCCT	CATCCTGCCGATGATATTCC	This study	
xsmad2	GAGAAGTGGTGCGAAAAAGC	GAAGCTGTAAAGGCCTGTGG	This study	
